# Direct quantification of in vivo mutagenesis and carcinogenesis using duplex sequencing

**DOI:** 10.1073/pnas.2013724117

**Published:** 2020-12-14

**Authors:** Charles C. Valentine, Robert R. Young, Mark R. Fielden, Rohan Kulkarni, Lindsey N. Williams, Tan Li, Sheroy Minocherhomji, Jesse J. Salk

**Affiliations:** ^a^TwinStrand Biosciences, Seattle, WA 98121;; ^b^MilliporeSigma/BioReliance Toxicology Testing Services, Rockville, MD 20850;; ^c^Amgen Research, Amgen, Thousand Oaks, CA 91320

**Keywords:** error-corrected sequencing, genotoxicity, genetic toxicology, preclinical cancer risk assessment, DNA repair

## Abstract

The ability to accurately measure mutations is critical for basic research and identifying potential drug and chemical carcinogens. Current methods for in vivo quantification of mutagenesis are limited because they rely on transgenic rodent systems that are low-throughput, expensive, prolonged, and do not fully represent other species such as humans. Next-generation sequencing (NGS) is a conceptually attractive alternative for detecting mutations in the DNA of any organism; however, the limit of resolution for standard NGS is poor. Technical error rates (∼1 × 10^−3^) of NGS obscure the true abundance of somatic mutations, which can exist at per-nucleotide frequencies ≤1 × 10^−7^. Using duplex sequencing, an extremely accurate error-corrected NGS (ecNGS) technology, we were able to detect mutations induced by three carcinogens in five tissues of two strains of mice within 31 d following exposure. We observed a strong correlation between mutation induction measured by duplex sequencing and the gold-standard transgenic rodent mutation assay. We identified exposure-specific mutation spectra of each compound through trinucleotide patterns of base substitution. We observed variation in mutation susceptibility by genomic region, as well as by DNA strand. We also identified a primordial marker of carcinogenesis in a cancer-predisposed strain of mice, as evidenced by clonal expansions of cells carrying an activated oncogene, less than a month after carcinogen exposure. These findings demonstrate that ecNGS is a powerful method for sensitively detecting and characterizing mutagenesis and the early clonal evolutionary hallmarks of carcinogenesis. Duplex sequencing can be broadly applied to basic mutational research, regulatory safety testing, and emerging clinical applications.

## Significance

Error-corrected next-generation sequencing (ecNGS) can be used to rapidly detect and quantify the in vivo mutagenic impact of environmental exposures or endogenous processes in any tissue, from any species, at any genomic location. The greater speed, higher scalability, richer data outputs, and cross-species and cross-locus applicability of ecNGS compared to existing methods make it a powerful new tool for mutational research, regulatory safety testing, and emerging clinical applications.

Carcinogenesis is rooted in somatic evolution. Cell populations bearing stochastically arising genetic mutations undergo iterative waves of natural selection that enrich for mutants which confer a phenotype of preferential survival or proliferation (1). The probability of cancer can be increased by carcinogens—exogenous exposures that either increase the abundance of mutations or facilitate a cell’s ability to proliferate upon selective pressures. Many chemicals induce DNA damage, thereby increasing the rate of potentially oncogenic DNA replication errors (2). The same is true for many forms of radiation (3). Nonmutagenic and nongenotoxic carcinogens act through a variety of secondary mechanisms such as inhibition of the immune system, cell-cycle overdrive to bypass normal DNA replication checkpoints, and induction of inflammation which may lead to both increased cellular proliferation and DNA damage, among others (4).

Preclinical genotoxicity and carcinogenicity testing of new compounds is often required before regulatory authority approval and subsequent human exposure (5, 6). However, current testing standards are slow and expensive; even in rodents, it takes years to reach the endpoint of tumor formation. Over the past 50 y, a variety of approaches have been developed to more quickly assess biomarkers of cancer risk by assaying DNA reactivity or mutagenic potential as surrogate endpoints for regulatory decision-making (7, 8). The most rapid and inexpensive of such methods include in vitro bacterial-based mutagenesis assays (e.g., the Ames test). Other in vitro and in vivo assays for mutation, chromosomal aberration induction, strand breakage, and formation of micronuclei are also available; however, their sensitivity and specificity for predicting human cancer risk is only modest.

In vivo, internationally accepted (5) mutagenesis assays using transgenic rodents (TGR) provide a powerful approximation of oncogenic risk, as they reflect whole-organism biology, but are also highly complex test systems (9). TGR mutagenesis assays require maintenance of multiple generations of animals bearing an artificial reporter gene, animal exposure to the test compound, euthanasia and necropsy several weeks after exposure, isolation of the integrated genetic reporter by phage packaging, and transfection of the phage into *Escherichia coli* for plaque counting on many Petri dishes under permissive and nonpermissive selection conditions to finally obtain a mutant frequency readout. Although effective, the infrastructure and expertise required for managing a protocol which carries host DNA through three kingdoms of life has hindered ubiquitous adoption.

Directly measuring ultrarare somatic mutations from extracted DNA while not being restricted by genomic locus, tissue, or organism (i.e., could be equally applied to rodents or humans) is appealing yet is currently impossible with conventional next-generation DNA sequencing (NGS). Standard NGS has a technical error rate (∼1 × 10^−3^) well above the true per-nucleotide mutant frequency of normal tissues (<1 × 10^−7^) (10). New technologies for error-corrected next generation sequencing (ecNGS) have shown great promise for low-frequency mutation detection in fields such as oncology and, conceptually, could be applied to genetic toxicology (11, 12). Duplex sequencing (DS), in particular, is an error-correction method that achieves extremely high accuracy by comparing reads derived from both original strands of DNA molecules to produce duplex consensus sequences that better represent the true sequence of the source DNA molecule. DS achieves a sensitivity and specificity several orders of magnitude greater than other methods that do not leverage paired-strand information; it is uniquely able to resolve mutants at the real-world frequencies produced by mutagens: on the order of 1 in 10 million (13).

In this study we tested the feasibility of using DS to measure the effect of genotoxicants in vivo. We assessed the DNA of two strains of mice which were treated with three different mutagenic carcinogens, each with distinct modes of action, and examined five different tissue types, to generate a total of almost 10 billion error-corrected nucleotides’ worth of data. In addition to comparing mutant frequencies with those obtained from a gold-standard TGR assay, we explored data types not possible with traditional assays, including mutant spectra, trinucleotide signatures, and variations in the relative mutagenicity around the genome. Our findings illustrate the richness of genotoxicity data that can be obtained directly from genomic DNA. Finally, we highlight a unique opportunity to apply ecNGS to drug and chemical safety testing for the concurrent detection of both mutagenesis and carcinogenesis.

## Results

### Experimental Overview.

**Fig. 1.**
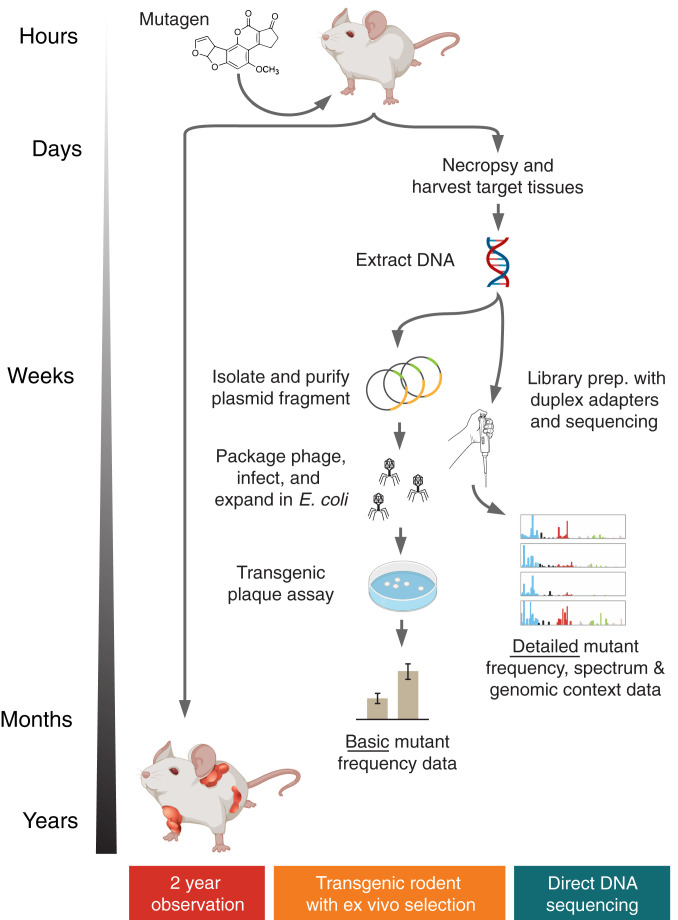
Approaches for in vivo carcinogenicity and mutagenicity testing. The gold standard for chemical cancer risk assessment involves exposing rodents to a test compound and assessing an increase in tumors relative to controls. While effective, the approach takes 2 y. Mutagenicity is one accepted surrogate for carcinogenicity risk which is more rapidly assessable. At present, TGR assays are the most validated in vivo mutagenesis measurement approach. A genetically modified strain carrying an artificial reporter gene is mutagen-exposed, necropsied, and the reporter is recovered from extracted DNA. Reporter DNA is then packaged into phage and transfected into bacteria for readout by counting total and mutation-bearing plaques following incubation at mutant-selectable temperature conditions. A simple mutant frequency can be determined within months, but the assay is complex. An ideal in vivo mutagenesis assay would be DNA sequencing-based and able to directly measure mutation abundance and spectrum in any tissue of any species without the need for genetic engineering or selection.

Current in vivo TGR mutagenesis detection assays measure the potential of a test article to generate mutations in a selectable reporter gene. Traditional 2-y carcinogenicity studies measure the ability of an agent to induce gross tumors in mice and rats. We designed two parallel mouse cohort studies to assess whether DS of genomic DNA could be used as an alternate method of quantifying both mutagenesis and early tumor-precursor formation (Fig. 1). We selected two transgenic strains of mice: Big Blue, a C56BL/6-derived strain bearing ∼40 integrated copies of lambda phage per cell, and Tg-rasH2, a cancer-predisposed strain carrying four copies of the human *HRAS* proto-oncogene (14, 15). The Big Blue mouse is one of the three most frequently used strains in the TGR mutagenesis assay and the Tg-rasH2 mouse is used for accelerated 6-mo carcinogenicity studies. Animals were dosed for up to 28 d with one of three mutagenic chemicals (or vehicle control, VC) before euthanasia and necropsy. Genomic DNA was isolated from various frozen tissues for subsequent mutational analysis (Table 1). The rationale for the selection of the specific strains, chemicals, tissues, and genic targets to be sequenced is detailed in the sections that follow.

**Table 1.** Summary of all samples along with cohort-level metadata

	Big Blue	Tg-rasH2	Total
Tissues (samples per group)	Liver (15)	Lung (10)	5 tissue types
	Marrow (17)	Spleen (10)	
		Blood (10)	
Treatment (samples per group)	B[α]P (10)	Urethane (15)	3 mutagens
	ENU (11)	VC (15)	
	VC (11)		
No. of samples	32	30	62 samples
Endogenous targets		*Ctnnb1*	7 endogenous targets
		*Hp*	
	*Ctnnb1*	*Hras*	
	*Hp*	*Kras*	
	*Polr1c*	*Nras*	
	*Rho*	*Polr1c*	
		*Rho*	
Transgenic targets	Lambda	Human *HRAS*	2 transgenic targets
	Bacteriophage		
	*cII*		
Duplex base pairs	4,716,990,836	4,923,565,684	9,640,556,520

### DS Yields Results Comparable to the Transgenic Rodent Assay.

We compared the frequencies of chemically induced mutations measured by a conventional plaque-based TGR assay (Big Blue) against those obtained by DS of the Big Blue reporter gene (*cII*) after isolation from mouse genomic DNA (gDNA) in the absence of any in vitro selection.

Eighteen Big Blue mice were treated with either VC (olive oil), benzo[α]pyrene (B[α]P) or *N*-ethyl-*N*-nitrosourea (ENU) for up to 28 d. We selected B[α]P and ENU based on their historical use as positive controls in many early studies of mutagenesis (9) and because they are recommended by Organisation of Economic Cooperation and Development Test Guideline (OECD TG) 488 for demonstration of proficiency at detecting in vivo mutagenesis with TGR assays (5). We evaluated bone marrow and liver tissue. The former was selected based on its high cell division rate and the latter based on its slower cell division rate and the presence of enzymes necessary for converting some nonreactive mutagenic compounds into their DNA-reactive metabolites. Corresponding plaque-based *cII* gene mutant frequency data using the Big Blue TGR assay were collected from all samples (*SI Appendix*, Table S1).

Genomic DNA was ultrasonically sheared and processed using a previously reported DS approach (16), which included enrichment for genic targets via hybrid capture (*SI Appendix*, Fig. S4). All samples were initially investigator-blinded with regard to treatment group. In this first experiment, we sequenced the multicopy *cII* transgene to a mean duplex depth (i.e., single duplex source molecule, deduplicated, coverage) of 39,668× per sample. DS mutant frequency per sample was calculated as the total number of unique nonreference nucleotides detected among all duplex reads of the *cII* gene divided by the total number of duplex base pairs of the *cII* gene sequenced.

**Fig. 2.**
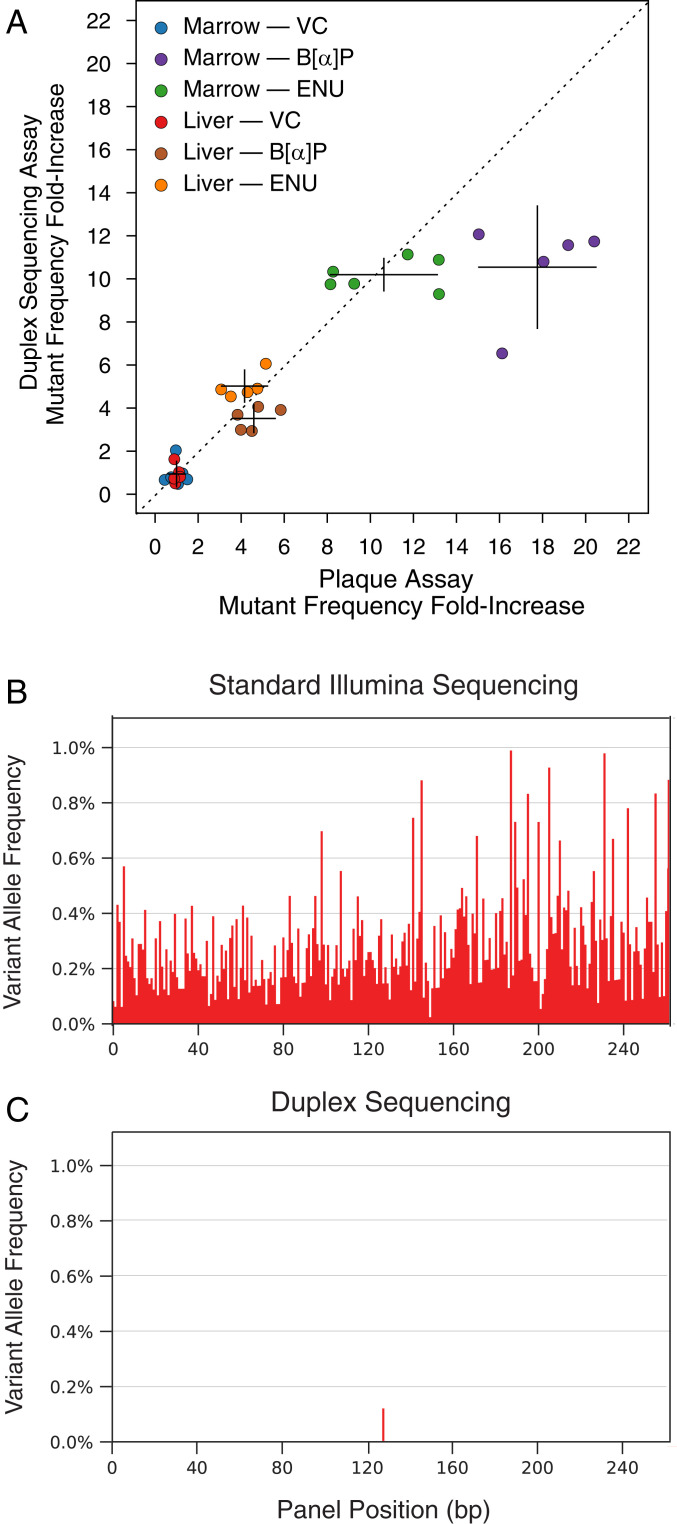
Comparison of DS and the TGR assay for quantifying in vivo chemical mutagenesis. (*A*) DS of a transgenic reporter gene (*cII*) relied upon by the Big Blue mouse TGR assay yields a similar fold induction of mutations in response to chemical mutagenesis as the readouts from the plaque-based assay. Error bars reflect the 95% CI. (*B*) Standard DNA sequencing has an error rate between 0.1% and 1% which obscures the presence of genuine low-frequency mutations. Shown are conventional NGS data from a representative 250-base pair (bp) section of the human *HRAS* transgene from the lung of a Tg-rasH2 mouse in the present study. Each bar corresponds to a nucleotide position. The height of each bar corresponds to the allele fraction of nonreference bases at that position when sequenced to >100,000× depth. Every position appears to be mutated at some frequency; nearly all of these are errors. (*C*) When the same sample is processed with DS, only a single authentic mutation remains.

The mean per-nucleotide mutant frequency measured by DS in the VC-, B[α]P-, and ENU-exposed groups was 1.48 × 10^−7^, 1.16 × 10^−6^ (7.84-fold increase over VC), and 1.27 × 10^−6^ (8.58-fold increase over VC), respectively. The mean fold increase detected between VC and mutagen-exposed groups was similar to that as measured by the conventional plaque assay, with per-gene mutant frequencies for VC, B[α]P, and ENU averaging 4.09 × 10^−5^, 4.42 × 10^−4^ (10.81 fold-increase over VC), and 3.06 × 10^−4^ (7.48-fold increase over VC), respectively (Fig. 2*A*). The extent of induction by both assays was dependent on the tissue type. Bone marrow cells, with their higher proliferation rate, accumulated mutations at 3.75 and 2.48 times the rate of the slower-dividing cells from the liver for B[α]P and ENU, respectively.

The extent of correlation between the fold change mutation induction of the two methods (*R*^2^ = 0.898) was encouraging given that the assays measure mutant frequency via two fundamentally different approaches. DS genotypes millions of unique nucleotides to assess the proportion that are mutated, whereas the plaque assay measures the proportion of phage-packaged *cII* genes that bear at least one mutation that sufficiently disrupts the function of the cII protein to result in phenotypic plaque formation. Put another way, mutations that are disruptive enough to prevent packaging or phage expression in *E. coli*, or those that are synonymous or otherwise have no functional impact on the cII protein, will not be scored.

One difference observed between the two methods was an attenuation of response to B[α]P in the marrow group by DS. This might be explained by an artificial skew due to the fold-increase calculation used, whereby slight variations in the frequency of VC will have disproportionately large effects on fold-increase measures but could also be wholly biological. It is conceivable that DNA adducts, or sites of true in vivo mismatches, could be artifactually “fixed” into double-stranded mutations when passaging reporter fragments through *E. coli* in the TGR assay, and that this effect is amplified as overall mutant frequency increases. DS, based in its fundamental error-correction principle, will not call adducted DNA bases as mutations when directly sequencing the *cII* genomic DNA, since a mutation has not yet formed on both strands of the DNA molecule.

Nevertheless, the overall correlation between DS and TGR assays was high and the mutant frequency measured in the VC samples by DS, on the order of 1 per 10 million mutant nucleotides sequenced, was 10,000-fold below the average technical error rate of standard NGS (Fig. 2 *B* and *C*). No difference in mutant frequency or spectrum between control and exposed samples could be detected when analyzing the data from either raw sequencing reads or ecNGS methods that do not account for complementary strand information (single-strand consensus sequencing) (*SI Appendix*, Fig. S1).

### DS Detects Similar Base Substitution Spectra between gDNA and Mutant Plaques in the TGR Assay.

The types of base substitution changes that are induced is an important element of mutagenesis testing. A lack of mutant frequency induction does not always mean a mutagen is nonmutagenic. Instead, analysis of the frequencies of specific transitions and transversions may reveal significant shifts in their relative contributions postexposure, indicating the mutagen is affecting the test system. Mutation spectra can also provide mechanistic insight into the nature of a mutagen. Although laborious, it is possible with plaque assays to characterize mutation spectra by picking and sequencing the clonal phage populations of many individual plaques or plaque pools (17, 18). Because mutations in plaques have been functionally selected, and the transgenic target is relatively small, it is possible that the spectral representation is skewed relative to a nonselection-based assay.

To assess whether mutation spectra are consistent between DS and TGR assays, we physically isolated, pooled, and sequenced (also with DS) 3,510 *cII* mutant plaques derived from Big Blue rodents exposed to VC, B[α]P, and ENU. We then compared the mutation spectra between the DS-analyzed mutant plaques and the DS-analyzed gDNA.

The base substitution spectra detected in the *cII* gene by both approaches were highly similar between methods (*P* > 0.999, χ^2^ test) (*SI Appendix*, Fig. S2) and yielded patterns consistent with expectations based on prior literature for both B[α]P (19, 20), an agent with reactive metabolites that intercalate DNA, similar to aflatoxin B_1_, and the alkylating agent ENU (21, 22). The majority of base substitutions observed following B[α]P exposure were characteristic G∙C→T∙A transversions (61.3% by DS, 57.0% by TGR), G∙C→C∙G transversions (17.5% by DS, 25.5% by TGR), and G∙C→A∙T transitions (16.2% by DS, 11.6% by TGR). The normally uncommon base substitutions with adenine or thymine as reference were increased in all ENU-exposed samples. The canonical transition that identifies ENU mutagenesis, C∙G→T∙A, was present at 32.2% by DS and 27.0% by TGR. These data add further weight of evidence that the mutations identified by DS reflect authentic biology and not technical artifacts.

### DS Detects Functional Classes of cII Mutants Undetected by the Plaque Assay.

The eponymously named TGR assays rely on a transgenic reporter cassette which can be recovered from genomic DNA. It is the ratio of mutant to wild-type genes, as inferred through phenotypically scoreable plaques, which permits the calculation of a mutant frequency (23–26). While these systems readily identify a subset of mutations in the reporter, others will not disrupt protein function and remain undetectable. Given that the primary use of TGR assays has been for relative, rather than absolute, mutational comparison between exposed and unexposed animals, the nonfunctional subset of mutants has historically been considered irrelevant.

Yet, with the increasing interest in more complex multinucleotide mutational spectra (27), the functional scoring of every base becomes essential given that a specific sequence may rarely, or never, occur in a small reporter region. DS does not have this limitation since there is no selection post-DNA extraction; all possible single-nucleotide variants (SNVs), multinucleotide variants, and indels can be equally well identified.

**Fig. 3.**
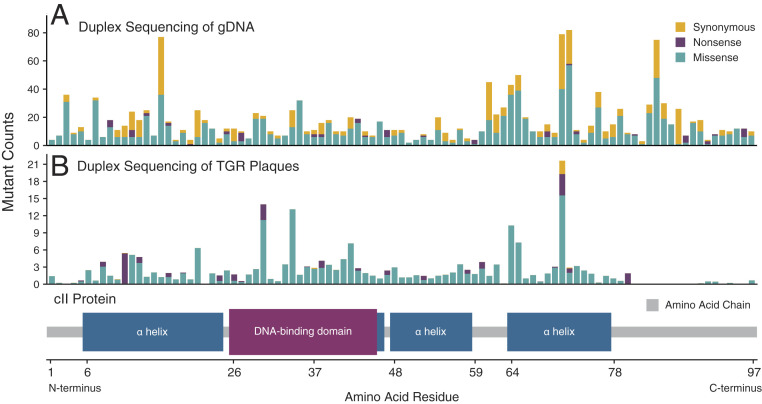
DS is agnostic to reporter gene function, whereas the TGR assay counts only phenotypically selectable mutations. (*A*) The distribution of all mutations identified by DS of *cII* from genomic DNA across all Big Blue tissues and treatment groups is shown by codon position and functional consequence. (*B*) The same analysis is presented for mutations identified from individually collected mutant plaques. Whereas DS recovers all functional classes of predicted amino acid mutations along the entire gene, mutations from picked mutant plaques that have lost a functional cII protein are devoid of synonymous variants and mutations at the nonessential C and N termini. Nucleotide positions with higher than average mutation counts by DS reflect mutagenic hotspots. The different mutation profile observed in the TGR plaque sequencing is more reflective of which sites are most phenotypically selected.

To illustrate the impact of TGR selection on mutant recovery, we visualized the functional class of all *cII* mutations identified by DS of either genomic DNA obtained directly from mouse samples (Fig. 3*A*) or from a pool of 3,510 individual mutant plaques that were isolated postselection (Fig. 3*B*). In the TGR plaque assay, the mutations were almost exclusively nonsense or missense across the entire 291 nucleotides of the *cII* gene (i.e., expected to result in the loss of *cII* protein function). Only a small number of synonymous base changes were identified, and these were always accompanied by a concomitant disruptive mutation elsewhere in the gene. Exceptionally few mutations were found at the N and C termini of the *cII* gene, presumably due to their lesser importance to protein function. In contrast, DS detected mutations of all functional classes at the expected nonsynonymous to synonymous (dN/dS) ratio along the entire length of the gene, including the termini regions.

### Rates of Chemical-Induced Mutagenesis Vary by Genomic Locus.

TGR assays rely on the assumption that the mutability of the *cII* lambda phage transgene is a representative surrogate for the entire mammalian genome. We hypothesized that local genomic features and functions of the genome such as transcriptional status, chromatin structure, and sequence context may modulate mutagenic sensitivity.

To test this idea, we used DS to measure the exposure-induced spectrum of mutations in four endogenous genes with different transcriptional status in different tissues: beta catenin (*Ctnnb1*), DNA-directed RNA polymerases I and III subunit RPAC1 (*Polr1c*), haptoglobin (*Hp*), and rhodopsin (*Rho*), as well as the *cII* transgene in Big Blue mouse liver and marrow of animals exposed to olive oil (VC), B[α]P, or ENU. We assessed mutations in the same four endogenous loci in the lung, spleen, and blood of Tg-rasH2 mice exposed to saline (VC) or urethane to investigate DS performance in a second mouse model.

**Fig. 4.**
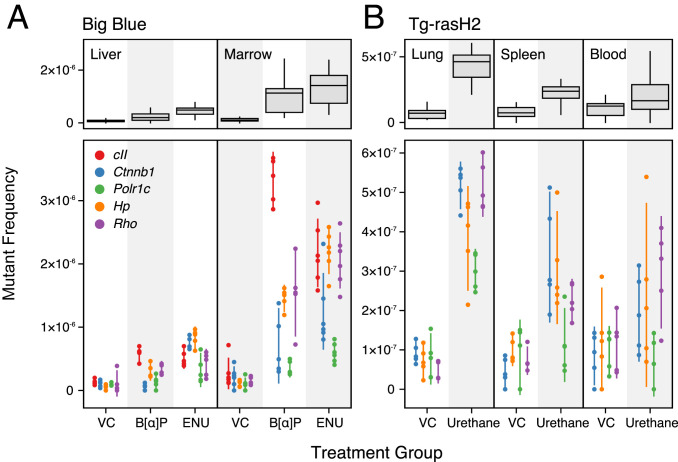
Sensitivity to mutagenesis varies by tissue type, mutagen, and genomic locus. SNV per-nucleotide mutant frequency (MF) is shown by tissue and treatment aggregated across all loci interrogated (*Top*) and by individual genic regions (*Bottom*). Box plots show all four quartiles of all data points for that tissue and treatment group. Scatter points show individual MF measurements from replicate animals in each cohort with line segments representing 95% CI. (*A*) Big Blue mouse study evaluating liver and bone marrow in animals exposed to VC, B[α]P, or ENU. (*B*) Tg-rasH2 mouse study evaluating lung, spleen, and peripheral blood in animals exposed to VC or urethane. There is no *cII* transgene in the Tg-rasH2 mouse model. Note the different *y* axis scaling between the two studies.

The DS SNV per-nucleotide mutant frequencies across mouse model, tissue, treatment group, and genomic locus are shown in Fig. 4. VC mutant frequencies averaged 1.14 × 10^−7^ in the Big Blue mouse model (Fig. 4*A*) and 9.03 × 10^−8^ in the Tg-rasH2 mouse model (Fig. 4*B*). The number of unique mutant nucleotides detected per VC sample ranged from 5 to 36 (mean 15.5) and were always nonzero (*SI Appendix*, Fig. S3). These frequencies are comparable to Chawanthayatham et al. (28), where a dimethyl sulfoxide (DMSO) vehicle-exposed transgenic *gptΔ* mouse was measured to have a mutant frequency of 2.7 × 10^−7^ in liver samples after DS of the reporter recovered from gDNA. We observed a mean background mutant frequency in the marrow (1.63 × 10^−7^) nearly twice that of peripheral blood (1.06 × 10^−7^), liver (9.63 × 10^−8^), lung (7.13 × 10^−8^), and spleen (7.45 × 10^−8^), which may relate to differences in relative cell-cycling times in these tissues.

In all mutagen-exposed samples, the mutant frequency was increased over the respective VC samples. However, the fold induction across tissue types varied considerably, as each compound has a different mutagenic potential, presumably related to varying physiologic factors such as tissue distribution, metabolism, and sensitivity to cell-turnover rate (29, 30).

The *cII* and *Rho* genes had highest mutant frequencies among all tested loci in bone marrow. Other genes, such as *Ctnnb1* and *Polr1c*, exhibited frequencies as much as eightfold lower. This disparity is potentially due to the differential impact of transcription levels and transcription-coupled repair (TCR) of lesions or local chromatin structure (31). *Ctnnb1* and *Polr1c* are thought to be transcribed in all tissues we tested, and therefore benefit from TCR, whereas *Rho* and *cII* are thought to be nontranscribed, and thus should not be impacted by TCR.

*Hp* was selected as a test gene because it is transcribed in the liver but not significantly in other tissues. The aforementioned logic cannot explain why *Hp* exhibited an elevated mutation rate compared to other genomic loci in the mouse liver. An additional genomic process related to the transcriptional status is DNA methylation. It is known that lesions on nucleotides immediately adjacent to a methylated cytosine have a lower probability of being repaired due to the relative bulk and proximal clustering of the adducts (32). This or other factors, such as differential base composition between sites, could also be at play.

Mechanisms aside, the widely variable mutant frequency we observe across different genomic loci indicates that no single locus is ever likely to be a comprehensive surrogate of the genome-wide impact of chemical mutation induction.

### Strand Bias of Mutations Reflects Functional Effects of the Genome.

To further investigate the potential role of TCR as a contributor to the observed differential regional sensitivity to mutagens, we examined the strandedness of mutations identified by DS at each locus. Mutational strand bias is defined as a difference in the relative propensity for a particular type of nucleotide change to occur on one DNA strand versus the other (e.g., A→C vs. T→G). This bias may result from multiple factors including transcription, epigenetic influences (e.g., methylation), proximity to replication origins, and nucleotide composition, among others (33, 34). We compared the per-nucleotide mutant frequency for each base substitution against its reciprocal substitution in our urethane-exposed mouse cohort. If a strand bias were to exist, then these frequencies would be unequal (35). We then correlated the extent of strand differences observed by genic region with predicted transcriptional status of each tissue.

Human transcription levels of four genes (*Ctnnb1*, *Polr1c*, *Hp*, and *Rho*) were used as a surrogate for those in mouse tissues and were obtained from the Genotype-Tissue Expression (GTEx) Project Portal (accessed on 2020-01-06). In humans, the levels of *Ctnnb1* expression are highest in lung (median transcripts per million [TPM] 164.4) and lower in spleen and blood (median TPM 100.3 and 25.75, respectively), whereas levels of *Polr1c* expression are low in all three (median TPM 19.27, 24.09, and 3.83, respectively). In humans, the genes *Hp* and *Rho* are largely nonexpressed in spleen, lung, and blood.

**Fig. 5.**
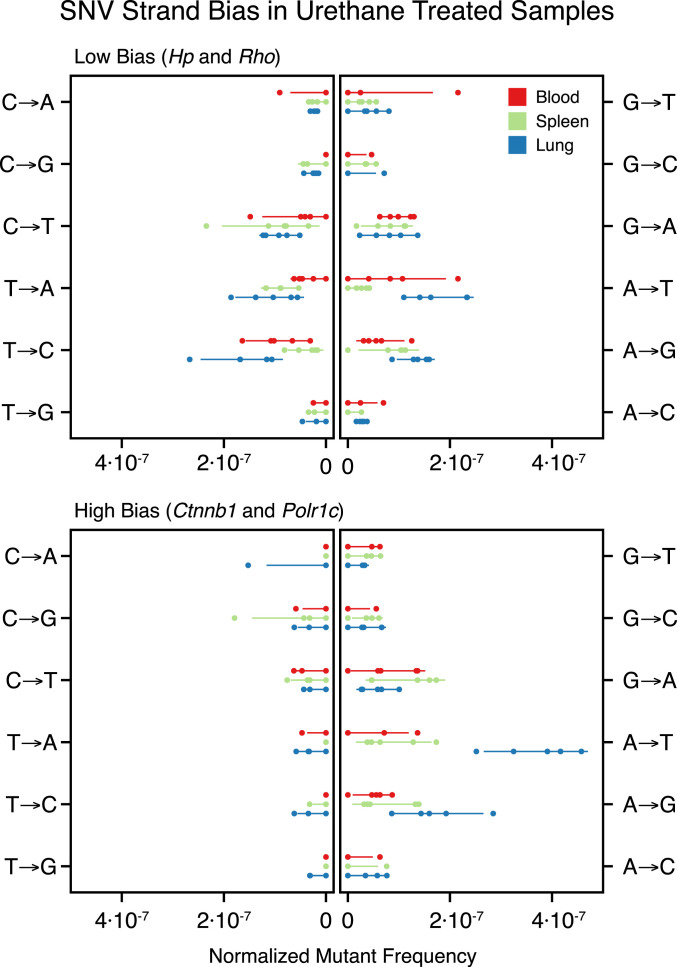
Strand bias in base substitutions exist in regions of moderate to high transcription. Mutational strand bias was seen in urethane exposed tissues for genes *Ctnnb1* and *Polr1c* (expressed in tissues examined) but not in *Hp* or *Rho* (not expressed in tissues examined). SNVs are normalized to the reference nucleotide in the forward direction of the transcribed strand. Individual replicates are shown with points, and 95% CI with line segments. Mutant frequencies were corrected for the nucleotide counts of each reference base in the target genes. The observed bias is evident in *Ctnnb1* and *Polr1c* as elevated frequencies of A→N and G→N variants relative to their complementary mutation (i.e., asymmetry around the vertical line), in contrast to the balanced spectrum of *Hp* and *Rho*. This difference is likely due to the mutation-attenuating effect of TCR on the template strand of transcribed regions of the genome.

Two genomic regions, *Ctnnb1* and *Polr1c*, showed high urethane-mediated strand bias (Fig. 5), which is consistent with a model of TCR since TCR predominantly repairs lesions on the transcribed strands of active genes (33). The majority of observed strand bias fell into two base substitution groups (T∙A→A∙T and T∙A→G∙C) in genes expressed in lung tissue (*Ctnnb1* and *Polr1c).* The mean reciprocal SNV fold difference of these mutation types across all tissue types was 11.6 and 9.0 in *Ctnnb1* and *Polr1c* versus 1.6 and 0.8 in (nontranscribed) *Hp* and *Rho*. The highest bias existed in lung tissue which is consistent with a TCR-related mechanism given that lung has the highest predicted transcription rate among the tissue types assayed.

### Unsupervised Clustering Resolves Simple Patterns of Mutagenesis.

**Fig. 6.**
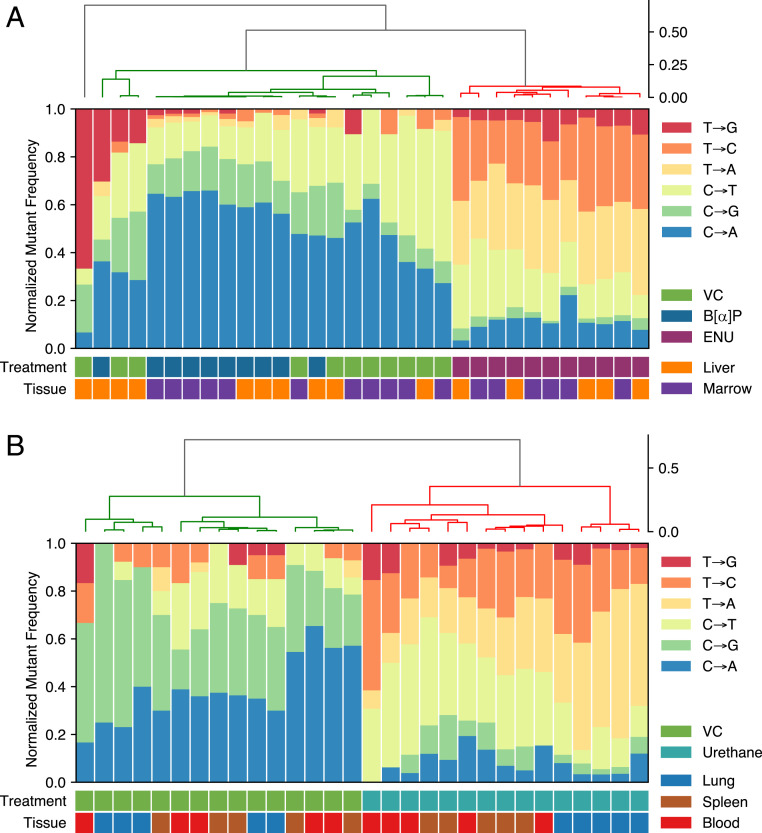
Unsupervised hierarchical clustering predicts mutagen treatment across samples. Clustering of simple spectrum probabilities was performed with the weighted (WGMA) method and cosine similarity metric. (*A*) Liver and marrow in Big Blue animals exposed to VC, ENU, or B[α]P. (*B*) Lung, spleen, and blood samples from the Tg-rasH2 cohort exposed to VC or urethane. Clustering was near-perfect except for distinguishing B[α]P from vehicle exposure in liver tissue where fewer mutational events were observed due to its lower proliferation rate.

We next sought to classify each sample into a mutagen class based solely on the simple spectrum of SNVs observed within the endogenous regions examined in both the Big Blue and Tg-rasH2 animals. The technique of unsupervised hierarchical clustering can resolve patterns of spectra as distinct clusters with common features (28). Fig. 6*A* shows a strong spectral distinction between ENU and both VC and B[α]P. However, the simple spectra of VC and B[α]P resolve poorly. A gradient of similarity is apparent in the VC and B[α]P cluster which suggests that, with deeper sequencing, it may be possible to fully resolve the two. No statistically valid clusters emerged that correlates with tissue type, suggesting that the patterns of mutagenesis for both B[α]P and ENU are similar in the liver and marrow of the Big Blue mouse. Fig. 6*B* shows perfect clustering by exposure due to the orthogonal patterns of urethane mutagenesis as compared to the unexposed tissues in Tg-rasH2 mice. We similarly saw no correlated clustering at the level of tissue type in Tg-rasH2 mice.

### Trinucleotide Spectrum of Treatment Groups Shows Distinct Patterns of Mutagenesis and Relates to Patterns Seen in Human Cancer.

**Fig. 7.**
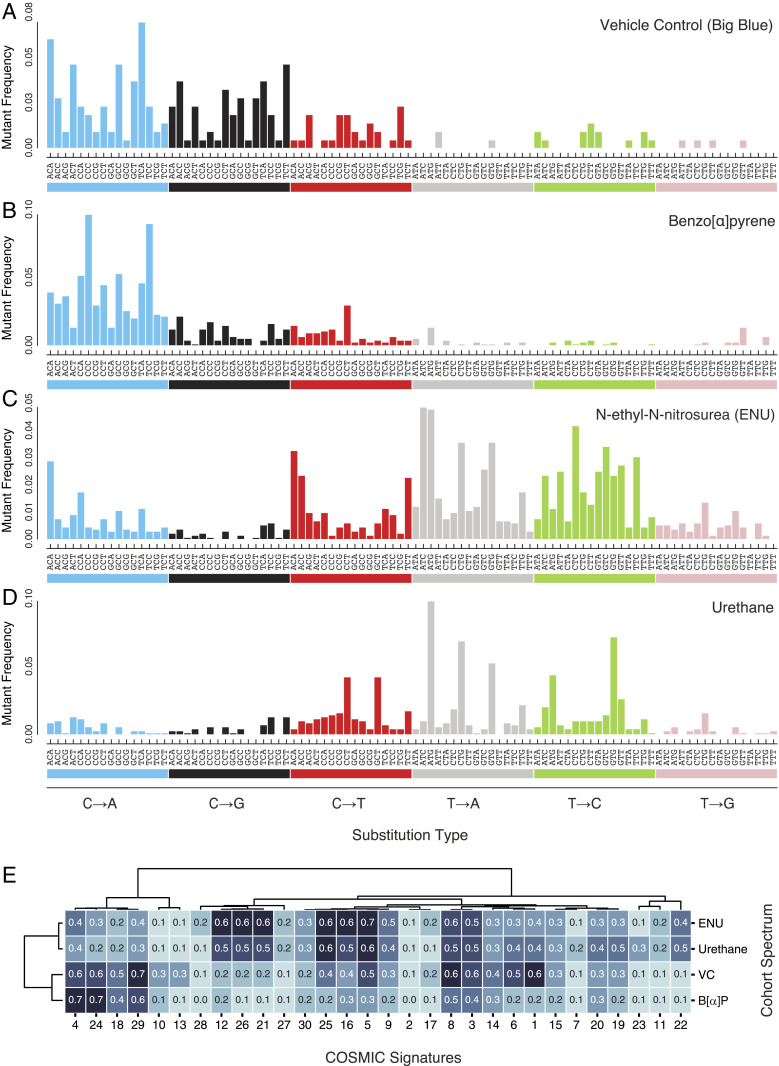
Trinucleotide base substitution spectra of each mutagen treatment reflects distinct mutational processes. The proportion of base substitutions in all trinucleotide contexts (pyrimidine notation) for the union of all endogenous mouse genic regions in (*A*) VC-, (*B*) B[α]P-, (*C*) ENU-, and (*D*) urethane-exposed mice. Each proportion was derived by normalizing the observed substitution types in each context by the relative abundance of that context in the regions examined. (*E*) Unsupervised hierarchical clustering of the first 30 published COSMIC signatures and the four cohort spectra. Clustering was performed with the weighted (WGMA) method and cosine similarity metric. B[α]P is most similar to Signatures 4, 24, and 29. Signature 24 is correlated with aflatoxin B1 exposure and has a similar mutagenic mode of action to the DNA intercalating reactive metabolites of B[α]P. VC is most like Signature 1, which is believed to reflect the age-associated mutagenic effect of reactive oxidative species and spontaneous deamination.

To further classify the patterns of SNVs by treatment group, we considered all possibilities of the 5′ and 3′ bases adjacent to the mutated base to create trinucleotide spectra (13, 28, 36). When enumerating all 96 possible SNVs within a unique trinucleotide context, a distinct pattern for each treatment group becomes apparent (Fig. 7 *A*–*D*) that show similarities to mutational signatures as extracted from thousands of human cancers (Fig. 7*E*).

The VC trinucleotide spectrum (Fig. 7*A*) is most similar to Signature 1 from the COSMIC catalog of somatic mutation signatures in human cancer (37) (cosine similarity of 0.6), identifiable through C∙G→T∙A transitions in CpG sites with a proposed etiology of unrepaired spontaneous deamination events at 5-methyl-cytosines. The most notable difference between the bulk trinucleotide spectrum of VC and Signature 1 is the extent of C∙G→A∙T and C∙G→G∙C transversions which most likely reflect endogenous oxidative damage, an age-related process (38).

The B[α]P trinucleotide spectrum (Fig. 7*B*) is predominantly driven by C∙G→A∙T mutations with a higher affinity for CpG sites. This observation is consistent with previous literature indicating that B[α]P adducts, when not repaired by TCR, lead to mutations most commonly found in sites of methylated CpG dinucleotides (32, 36). This spectrum is highly similar to Signature 4 (0.7 cosine similarity) and Signature 29 (0.6 cosine similarity), both of which have proposed etiologies of human exposure to tobacco where B[α]P and other polycyclic aromatic hydrocarbons are major mutagenic carcinogens. The spectrum for in vivo murine exposure to B[α]P is equally comparable to Signature 4 and Signature 24 (0.7 cosine similarity), likely due to similar mutagenic modes of action between B[α]P and aflatoxin (the proposed etiology of signature 24) (28).

The urethane trinucleotide spectrum (Fig. 7*D*) has no confidently assignable analog in the COSMIC signature set. As compared to the simple spectrum of urethane in Fig. 6*B*, a periodic pattern of T∙A→A∙T in 5′-NTG-3′ emerges. This pattern of highly residue-specific mutagenicity has been previously observed in the trinucleotide spectra of whole-genome sequencing data from adenomas of urethane-exposed mice (39) as well as in urethane-exposed lung tissue of mice weeks after exposure, as recently detected by another ecNGS method (40).

### Oncogenic Ras Mutations Undergo Strong In Vivo Selection within Weeks of Carcinogen Exposure in Cancer-Prone Tg-rasH2 Mice.

The Tg-rasH2 mouse model contains four tandem copies of human *HRAS* with an activating enhancer mutation to boost oncogene expression (14). The combination of enhanced transcription and increased proto-oncogene copy number predisposes the strain to cancer. Use of these mice in a 6-mo cancer bioassay is accepted under International Council for Harmonisation (ICH) S1B guidelines as an accelerated substitute for the traditional 2-y mouse cancer bioassay used for pharmaceutical safety assessment (41). Exposure to urethane, a commonly used positive control mutagen, results in splenic hemangiosarcomas and lung adenocarcinomas in nearly all animals by 10 wk postexposure.

**Fig. 8.**
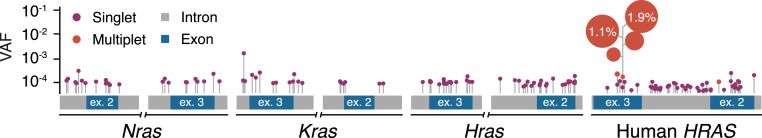
Early neoplastic evolution in cancer-prone mice following carcinogen exposure. The location and variant allele frequency (VAF) of SNVs are visualized across the genomic intervals for the introns and exons captured from the endogenous mouse *Ras* family of genes as well as the human transgenic *HRAS* loci from the Tg-rasH2 mouse model. Singlets are mutations identified in a single molecule of a sample. Multiplets are an identical mutation identified within multiple molecules within the same sample and may represent a clonal expansion event. Pooled data from all tissues in the experiment (lung, spleen, and blood) are included. The height of each point (log scale) corresponds to the VAF of each SNV. The size of the point corresponds to the number of counts observed for the mutant allele. A cluster of multiplet A∙T→T∙A transversions at the human oncogenic *HRAS* codon 61 hotspot is seen in four out of five urethane-exposed lung samples and one out of five urethane-exposed splenic samples (*SI Appendix*, Table S4). The observation of an identical mutation in independent samples with high-frequency multiplets in a well-established cancer driver gene likely indicates positive selection. Notably, these clones are defined by the transversion A∙T→T∙A in the context NTG which is characteristic of urethane mutagenesis.

We examined the effect of urethane exposure on the *HRAS* transgene, as well as the endogenous *Hras*, *Kras*, and *Nras* genes, at DNA residues most commonly mutated in human cancers (Fig. 8).

In contrast to the endogenous *Ras* family genes, the human *HRAS* transgene is present in four copies per haploid genome—each under the control of a tandem promoter and enhancer, but without the repression system that is present at the endogenous human *HRAS* locus. We postulated that the mechanism of activation of human *HRAS* in the Tg-rasH2 model would positively influence selection of the cells harboring the activating mutations and would be observable as outgrowth of clones bearing mutations at hotspot residues relative to residues not under positive selection. Indeed, we observed compelling signs of selection as evidenced by focally high variant allele frequencies (VAFs) of activating mutations at the canonical codon 61 hotspot in exon 3 in the human *HRAS* transgene, but not at other sites in that gene, nor at homologous sites in the endogenous mouse *Ras* family. Sizable clonal expansions of this mutation were detected in four out of five lung samples, one out of five spleen samples, and in no blood samples, which is consistent with the historically known relative frequency of tumors in each tissue.

Moreover, not only are the variant allele frequencies as much as 100-fold higher than seen for any other endogenous gene variant but the absolute counts of mutant alleles at this locus is very high (>5), which offers strong statistical support for these clones existing as authentic expansions and not as independent mutated residues occurring by chance (*SI Appendix*, Table S4). Notably, all clonal mutations observed at codon 61 are A∙T→T∙A transversions in the context 5′-CTG-3′, which conforms to the context 5′-NTG-3′, which is highly mutated across all genes in the urethane-exposed mouse samples (39) (Fig. 7*D*). Other types of mutations at codon 61 could lead to the same amino acid change, so the combination of the specific nucleotide substitution observed, the clone size relative to that of other loci, and the repeated observation across independent samples of the most tumor prone tissues paints a comprehensive picture of both a urethane-mediated mutagenic trigger and a carcinogenic process that follows.

## Discussion

We have demonstrated that DS, an extremely accurate ecNGS method, is a powerful tool for the field of genetic toxicology that can be used to assess both mutagenesis and carcinogenesis in vivo. Unlike conventional in vivo mutagenesis assays, DS does not rely on selection but rather on unbiased digital counting of billions of individual nucleotides directly from the DNA region of interest. This yields data that are both richer and more broadly representative of the genome than current tools and allows fundamentally new types of biological questions to be asked.

From sequence data it is possible to mine a wealth of information including mutation spectrum, trinucleotide mutation signatures, and predicted functional consequences of mutations. By virtue of not being limited to a specific reporter, we showed that the relative susceptibility to chemical mutagenesis varies significantly by genomic locus and is further influenced by tissue. We could infer this to be (at least partially) the result of nonuniform TCR, as evidenced by the consistent asymmetry of certain mutation types between transcribed and nontranscribed strands. The examples shown here are limited by the modest number of loci and tissues, the inference of transcriptional status based on another species, and can be improved upon in future studies. It is likely that many other factors beyond transcriptional status shape the relative plasticity of the genome and can be uncovered with careful investigations.

The ability to directly observe subtle regional mutant frequency differences, on the order of 1 in 10 million, is extraordinary in terms of biological study opportunities but also raises practical questions for regulatory usage. For example, what would define the optimal subset of the genome to be used for drug and chemical safety testing? For some applications, a diverse, genome-representative panel makes the most sense; for others it might be preferable to enrich for regions that are predisposed to certain mutagenic processes (42) or have unique repair biology (35).

Not all carcinogens are mutagens. Drugs and chemicals which are not mutagenic will not produce a signal in mutagenesis assays—either conventional or sequencing-based. However, as shown here, it appears possible to use ecNGS to infer carcinogenesis via detection of clonal expansions carrying oncogenic driver mutations as a marker of a neoplastic phenotype (43). This concept is more complex to design, insofar as it necessitates some a priori knowledge about the common drivers that are operative in different tissues in response to different classes of carcinogens. However, there is simply no other approach, convenient or not, that can quantitate these signals in less than a month from exposure. The proof-of-concept illustrated here relied on a mutagenic chemical in a cancer-predisposed mouse strain; future efforts will be needed to demonstrate the same with nongenotoxic carcinogens in wild-type animals.

A further advantage of ecNGS is the breadth of applicability, in vivo or in vitro, to any tissue from any species. In vivo selection-based assays are organism- and reporter-specific; the former restricts testing to rodents, and the latter confers potential biases to mutational spectrum and does not allow targeting of specific genomic regions. The only in vivo mutagenesis assay that does not depend on in vitro selection, the Pig-a Gene Mutation Assay, classically restricted to only erythrocytes, requires bioavailability to the bone marrow compartment, cannot be used for spectrum analysis, and necessitates access to flow cytometry equipment (44). In contrast, next-generation DNA sequencing platforms are widely available and can be automated to handle thousands of samples per day, thus rendering the approach tractable for many different types of laboratories.

We are not the first to apply NGS to mutagenesis applications (13). Sequencing the reporter gene from pooled clones from TGRs has been used to identify in vivo mutagenic signatures (17). Single-cell cloning of mutagen-exposed cultured cells and patient-derived organoids has been used to identify in vitro and in vivo mutagenic signatures (45–48). In each case, cloning, followed by biological amplification, was required to resolve single-cell mutational signals, which would otherwise be undetectable in a background of sequencing errors. We have previously used DS to measure trinucleotide signatures in phage-recovered reporter DNA of mutagen-exposed transgenic mice without the need for cloning (28). Others have characterized mutational spectra directly from human DNA using a form of very-low-depth whole-genome DS without added molecular tags (49). However, each of these methods has factors that limit its practicality for broad usability.

The cost of any NGS-based technique is an important consideration, particularly when compared to something as routine as the bacterial Ames assay. DS further multiplies sequencing costs because of the need for redundant copies of each source strand as a part of the consensus-based error correction strategy. However, over the last 12 y, the cost of NGS has fallen nearly four orders of magnitude, whereas the cost of conventional genetic toxicology assays has remained largely unchanged. Extrapolating forward, we anticipate that equipoise will be reached. Savings by virtue of not needing to breed genetically engineered animals, the ability to repurpose tissue or cells already generated for other assays (supporting the 3R concept of replacement, reduction, and refinement), decreased labor, and greater automatability should also serve to increase efficiencies and lessen animal use (50).

Beyond being undesirable, animal testing is simply not possible for some applications. New forms of mutagenesis, such as CRISPR-Cas and other gene editing technologies, are highly sequence-specific and cannot be easily derisked in alternative genomes or using reporter genes (51, 52). Being able to carry out rapid in-human genotoxicity assessment as a part of early clinical trials may also be important for applications where there is urgency to develop therapies, such as drugs being tested against the 2019 pandemic coronavirus (53) and those needed in future public health emergencies.

Controlled drug and chemical safety testing are not the only reasons to screen for mutagenic and carcinogenic processes. Humans are inadvertently exposed to many environmental carcinogens (54, 55). The ability to identify biomarkers of mutagenic exposures using DNA from tissue or noninvasive samples such as blood, urine, or saliva is an opportunity for managing individual patients via risk-stratified cancer screening efforts as well as public health surveillance to facilitate carcinogenic source control (13). Deeper investigations into human cancer clusters (56), monitoring those at risk for occupational carcinogenic exposures (57), such as firefighters (58) and astronauts (59), and surveilling the genomes of sentinel species in the environment as first-alarm biosensors (60) are all made possible when DNA can be analyzed directly.

Almost four decades have passed since it was envisioned that the entirety of one’s exposure history might be gleaned from a single drop of blood (61). While this remains a lofty ambition, the data we have shown here suggest that it is not wholly implausible. Our work indicates that there is a much greater amount of information recorded in the somatic genome than we have previously been able to appreciate or access. Future studies are needed to determine how best to capitalize on this data for basic research applications, preclinical safety testing, and in-human studies.

## Materials and Methods

### Animal Treatment and Tissue Collection.

All animals used in this study were housed at Association for Assessment and Accreditation of Laboratory Animal Care International–accredited facilities and all research protocols were approved by these facilities respective to their Institutional Animal Care and Use Committees.

Big Blue C57BL/6 homozygous male mice [C57BL/6-Tg(TacLIZa)A1Jsh] bred by Taconic Biosciences on behalf of BioReliance were dosed daily by oral gavage with 5 mL/kg VC (olive oil) or B[α]P formulated in the vehicle at a dose level of 50 mg/kg per day for 28 d. A third cohort of Big Blue mice were dosed by oral gavage with 40 mg/kg per day (10 mL/kg) of ENU formulated in phosphate buffer solution (pH 6.0) on days 1, 2, and 3. All animals were necropsied on study day 31.

Tg-rasH2 male mice [CByB6F1-Tg(HRAS)2Jic] from Taconic Biosciences received a total of three intraperitoneal injections of VC (saline) or urethane (1,000 mg/kg per injection) at a dose volume of 10 mL/kg per injection on days 1, 3, and 5. Animals were necropsied on study day 29.

Liver, lung, and spleen samples were collected and then flash-frozen. Bone marrow was flushed from femurs with saline and centrifuged, and the resulting pellet was flash-frozen. Blood was collected in K2 ethylenediaminetetraacetic acid (EDTA) tubes and flash-frozen.

Studies were generally consistent with OECD TG 488 guidelines except that ENU and urethane were dosed less than daily but at a frequency known to produce systemic mutagenic exposures. The sampling time for the urethane study was at day 29 and not day 31.

### Plaque Assay for Mutant Analysis.

High-molecular-weight DNA was isolated from frozen Big Blue and Tg-rasH2 tissues using methods as described in the RecoverEase product use manual Rev. B (720202; Agilent). Vector recovery from genomic DNA, vector packaging into infectious lambda phage particles, and plating for mutant analysis was performed using methods described in the λ Select-cII Mutation Detection System for Big Blue Rodents product use manual Rev. A (720120; Agilent) (5).

### Phage and Mouse DNA for Duplex Sequencing.

Phage DNA was purified from phage plaques punched from the *E. coli* lawn on agar mutant selection plates following 2 d of incubation at 24 °C. Agar plugs were pooled by mutagen treatment group in SM buffer and then frozen for storage. DNA was purified using the QIAEX II Gel Extraction Kit (20021; Qiagen). Mouse genomic DNA was purified from liver, bone marrow, lung, spleen, and blood. Approximately 3- × 3- × 3-mm tissue sections were pulverized with a disposable tube pestle in a microfuge tube. DNA was extracted using the Qiagen DNeasy Blood and Tissue Kit (69504; Qiagen).

### Duplex Sequencing.

Extracted genomic DNA was ultrasonically sheared to a median fragment size of ∼300 base pairs using a Covaris system. Sheared DNA was further processed using a prototype mixture of enzymes with glycosylase and lyase activity for the purpose of excising certain forms of DNA damage and cleaving phosphodiester backbones at resulting abasic sites to render damaged or incomplete duplex templates unamplifiable (TwinStrand Biosciences). DNA was end-polished, A-tailed, and ligated to DS adapters containing semidegenerate unique molecular identifies (TwinStrand Biosciences) via the general method described previously (10, 16). Adapter-ligated DNA fragments were then PCR-amplified with primers containing dual unique indexes. After the initial PCR, samples were individually subjected to tandem hybrid capture using 120-mer 5' biotinylated DNA oligo probes (Integrated DNA Technologies), for a total of two captures. The first (indexing) and second PCR respectively entailed 10 and 14 cycles. The third PCR involved a variable number of cycles until the library could be accurately quantified. Resulting libraries were quantified, pooled, and sequenced on an Illumina NextSeq 500 using 151-base pair paired-end reads with vendor-supplied reagents. Where necessary, SYBR-based qPCR was used to determine appropriate DNA input by normalizing phage and mouse DNA across library preparations by total genome equivalents. Library input, before shearing, of plaque DNA was ∼100 pg and the genomic DNA input for all mouse samples was ∼500 ng. A summary of sequencing data yields for Big Blue and Tg-rasH2 samples is listed in *SI Appendix*, Tables S2 and S3.

### Hybrid Selection Panel Design.

Hybrid selection baits for all targets were designed to intentionally avoid capturing any nucleotide sequence within 10 base pairs of a repeat-masked interval as defined in RepBase (*SI Appendix*, Fig. S4) (62). Intronic regions adjacent to the exons of the target genes were baited to provide a functionally neutral and noncoding view on the pressures of mutagenesis near exonic targets. Duplex consensus base pairs and subsequent variant calls were only reported over a region defined by the same repeat-mask rule as for the bait target design. All libraries achieved 99.9% alignment of duplex consensus bases over the target territories with less than 0.001% of off-panel alignment. All targets were of expected uniform coverage given that no off-target alignment to pseudogenes or repetitive genomic sequences was observed.

Baits were also designed to target the *cII* transgene in the Big Blue mouse model and the human *HRAS* transgene in the Tg-rasH2 mouse model. The multicopy *cII* transgene was sequenced to a median target coverage of 39,668× and the multicopy human *HRAS* transgene to 9,012×.

### Consensus Calling and Consensus Postprocessing.

Consensus calling was carried out as generally described in “Calling Duplex Consensus Reads” from the Fulcrum Genomics fgbio tool suite (63). The algorithm proceeds with aligning the raw reads with bwa. After alignment, read pairs were grouped based on the corrected unique molecular identifier nucleotide bases and their shear point pair as determined through primary mapping coordinates. The read pairs within their read pair groups were then unmapped and oriented into the direction they were in as outputted from the sequencing instrument. Quality trimming using a running-sum algorithm was used to eliminate poor-quality three-prime sequence. Bases with low quality were masked to “N” for an ambiguous base assignment. Cigar filtering and cigar grouping was performed within each read pair group to help mitigate the poisoning effect of artifactual indels in individual reads introduced in library preparation or sequencing. Finally, consensus reads were created, from which duplex consensus reads meeting prespecified confidence criteria were filtered. Barcode error correction was performed using a known whitelist of barcodes, a maximum number of mismatches between a barcode and an expected barcode of 1, and a minimum Hamming distance to the next most likely known barcode of 2. After duplex consensus calling, the read pairs underwent balanced overlap hard clipping to eliminate biases from double counting bases due to duplicate observation within an overlapping paired-end read. Duplex consensus reads were then end-trimmed and interspecies decontamination was performed using a *k*-mer–based taxonomic classifier (*SI Appendix*).

### Variant Calling and Variant Interpretation.

Variants were called using VarDictJava with all parameters optimized to collect variants of any alternate allelic count greater than, or equal to, one (64).

There are two polar interpretations one can make when an identical canonical variant is observed multiple times in the same sample. The first assumption is that the observations were independent and that they were acquired during unrelated episodes in multiple independent cells and are not the product of a clonal expansion and shared cell lineage. The second assumption is that the alternate allele observations are a clonal expansion of a single mutagenic event and can all be attributed to one initial mutagenic event.

When classifying variant calls as either independent observations, or from a clonal origin, we first fit a log-normal distribution to all variants that were not germline. Any outliers to this distribution with multiple observations are deemed to have arisen from a single origin. This method may serve to undercount multiple independent mutations at the same site under extraordinary specific mutagenic conditions. For example, the clonally expanded A∙T→T∙A transversion at codon 61 in the *HRAS* transgene was a significant outlier to this model and was highly correlated with urethane exposure. The VAF of these expanded mutations varied 100× in urethane-exposed lung tissues, however, our calculation of per-nucleotide mutant frequency varied only ∼2×, indicating that this one tissue-specific residue was under the highest selective pressures for expansion beyond any other residue in any other tissue within the panel territory.

### Hierarchical Clustering of Base Substitution Spectra.

All clustering was performed using the Wald method and the cosine distance metric. Leaves were ordered based on a fast-optimal ordering algorithm (65). Simple base substitution spectrum clustering was achieved by first converting all base substitutions into pyrimidine space and then normalizing by the frequencies of nucleotides in the target region. Clustering of trinucleotide spectra was achieved in a similar manner where base substitutions were converted into pyrimidine space and then partitioned into 16 categories based on all of the combinations of five and three prime adjacent bases (37). Subsequent normalization of trinucleotide spectra was performed using the frequencies of 3-mers in the target regions.

## Supplementary Material

Supplementary File

Supplementary File

Supplementary File

## Data Availability

Final filtered and decontaminated error-corrected alignments for all 62 mouse samples in the BAM file format are deposited in the Sequence Read Archive under BioProject accession no. PRJNA673916 (66).
