# Supplementary material for: Direct quantification of in vivo mutagenesis and carcinogenesis using duplex sequencing
Source: Proc Natl Acad Sci U S A. 2020 Dec 14;117(52):33414–25. doi: 10.1073/pnas.2013724117 (PMC7776782; doi:10.1073/pnas.2013724117)
Supplement: Supplementary File [file pnas.2013724117.sapp.pdf]

SUPPLEMENTARY INFORMATION APPENDIX

Inter-species contamination is robustly detected by Duplex Sequencing

A consequence of extremely accurate error-correction next generation sequencing (ecNGS) technologies is the detection of ultra-rare intra-species contamination and how false positive alignments of those sequencing reads can bias per-nucleotide mutant frequency (MF) calculation by more than 100-fold. The false positive alignment of short reads not from the target species is particularly likely when sample processing is done near samples that are of an alternate species. This issue is exacerbated when targeting regions of high homology among all species, such as in conserved or exonic regions of the genome (**SI Appendix, Fig. S5**).

The solution we developed for handling inter-species read-pair decontamination relies on taxonomic classification of all error-corrected sequences from the entire study to ensure only the read pairs that match the target species with high confidence are kept for downstream analysis.

A taxonomy database was constructed with *k*-mers from human, rat, cow, and mouse. The taxonomic classifier Kraken [1] was used to identify error-corrected paired-end contaminating reads, as well as confidently indicating which reads were only from *Mus musculus* origin. Reads that are left unassigned due to this method are often true sequences from the source genomes, however, they contain an `N`-call or variant base often enough such that a single *k*-mer cannot exist that indicates a positive classification to the target genome. Reads of ambiguous assignment were discarded as they did not contain enough sequence information to positively assign them to any of the organisms at the species level.

To eliminate confounding assignment due to the human *HRAS* transgene in the Tg-rasH2 mouse model, a masked human genome was used for all classification where the mask territory was the exact sequence copy as integrated into Tg-rasH2.

Out of a total of 52,509,726 error-corrected paired-end reads across all 62 (1.2×10^{-4}%) murine tissue samples, 50,910,333 were taxonomically classified as *Mus musculus*, 34 to *Rattus norvegicus albus*, 33 (6.3×10^{-4}%) to *Homo sapiens*, and 0 to *Bos taurus* (0%). Exactly 84,865 (0.2%) paired-end reads were unclassified and 1,514,494 (2.8%) were from an ambiguous taxonomic origin. Only sequence data that could be positively identified as originating from the mouse genome was reserved for downstream analysis. Furthermore, every error-corrected paired-end read supporting a variant call in this cohort underwent manual review and BLAST+ alignment using the Blast nucleotide (nt) collection to confirm the true positive rate of taxonomic classification on this error-corrected dataset as being a perfect 100.000000%.

Tissue samples from vehicle control exposed mouse ID 9951 contained 29 paired-end reads from *Homo sapiens* and a tissue sample from the benzo[α]pyrene exposed mouse ID 9310 contained 28 paired-end reads from *Rattus norvegicus albus* suggesting that most contaminating events in both mouse cohorts were punctuated and private to just a few samples. The mean per-nucleotide mutant frequency for mouse 9951 is 1.2×10^{-7} and if contaminating reads were not removed, the mean per-nucleotide mutant frequency would have risen to a rate equivalent, or greater than, the mutant frequencies detected in the positive control samples.

SUPPLEMENTARY FIGURES

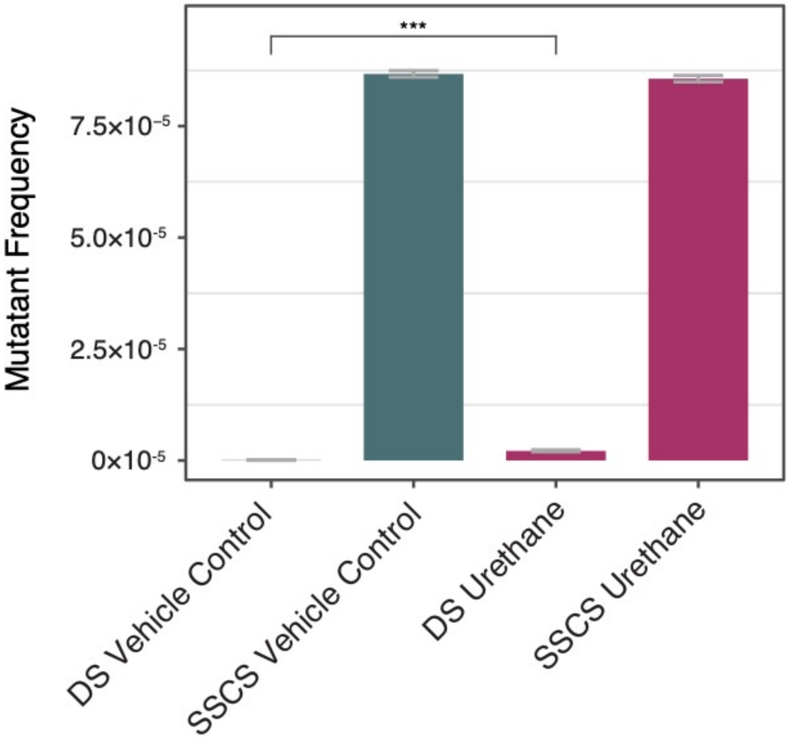

Figure S1. MF comparison in a mutagen exposed sample with and without duplex consensus level error-correction. Alternative forms of error-corrected next generation sequencing (ecNGS) may perform the error-correction on single-strands without resolving a complete duplex consensus. These single-strand error-correction forms of ecNGS are not sensitive enough for resolving small effect sizes in mutant frequency induction from experiments like those in the TGR assays. To illustrate this, we performed single-strand error-correction data using Duplex Sequencing Adapters on two Tg-rasH2 mouse lung samples, one treated with urethane and one treated with the vehicle control. The per-nucleotide mutant frequencies for the vehicle control and urethane-exposed samples are 8.2×10^{-8} and 2.15×10^{-6} using Duplex Sequencing. When measuring the same metric using only single-strand consensus sequencing (SSCS), the two mutant frequencies rise to 8.6×10^{-5} and 8.6×10^{-5}, respectively. The difference between the mutant frequencies of the exposed and control tissues using Duplex Sequencing are different with a p-value less than 2.2×10^{-16}. This is in contrast to the single-strand error-correction measurements of mutant frequency which are not significant (p-value 0.98). Both statistical tests were performed using the Fisher's exact test for count data. Error bars reflect 95% confidence intervals.

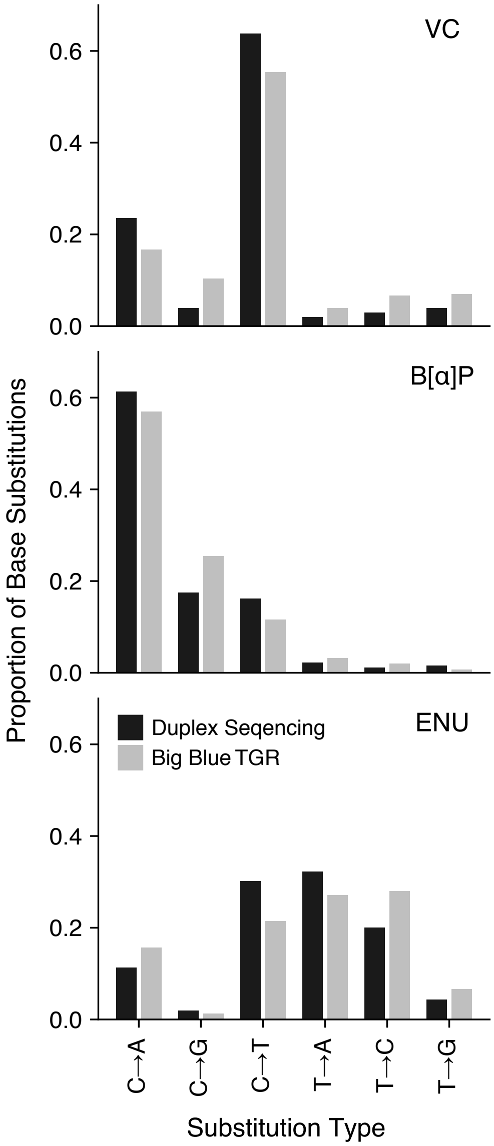

Figure S2. Mutation spectra observed by Duplex Sequencing of genomic DNA and individually sampled mutant plaques from the TGR assay are equivalent. The proportion of single nucleotide variants (SNV) within the *cII* gene are shown for individually picked mutant phage plaques produced from Big Blue rodent tissue and Duplex Sequencing of the *cII* transgene directly from gDNA. SNVs are designated with pyrimidine as the reference. The two methods yield the same spectrum for all treatment groups (p-values >0.999, chi-squared test). Proportions were calculated by dividing the total observations of SNVs by the total number of duplex bases within the *cII* interval and normalizing to one.

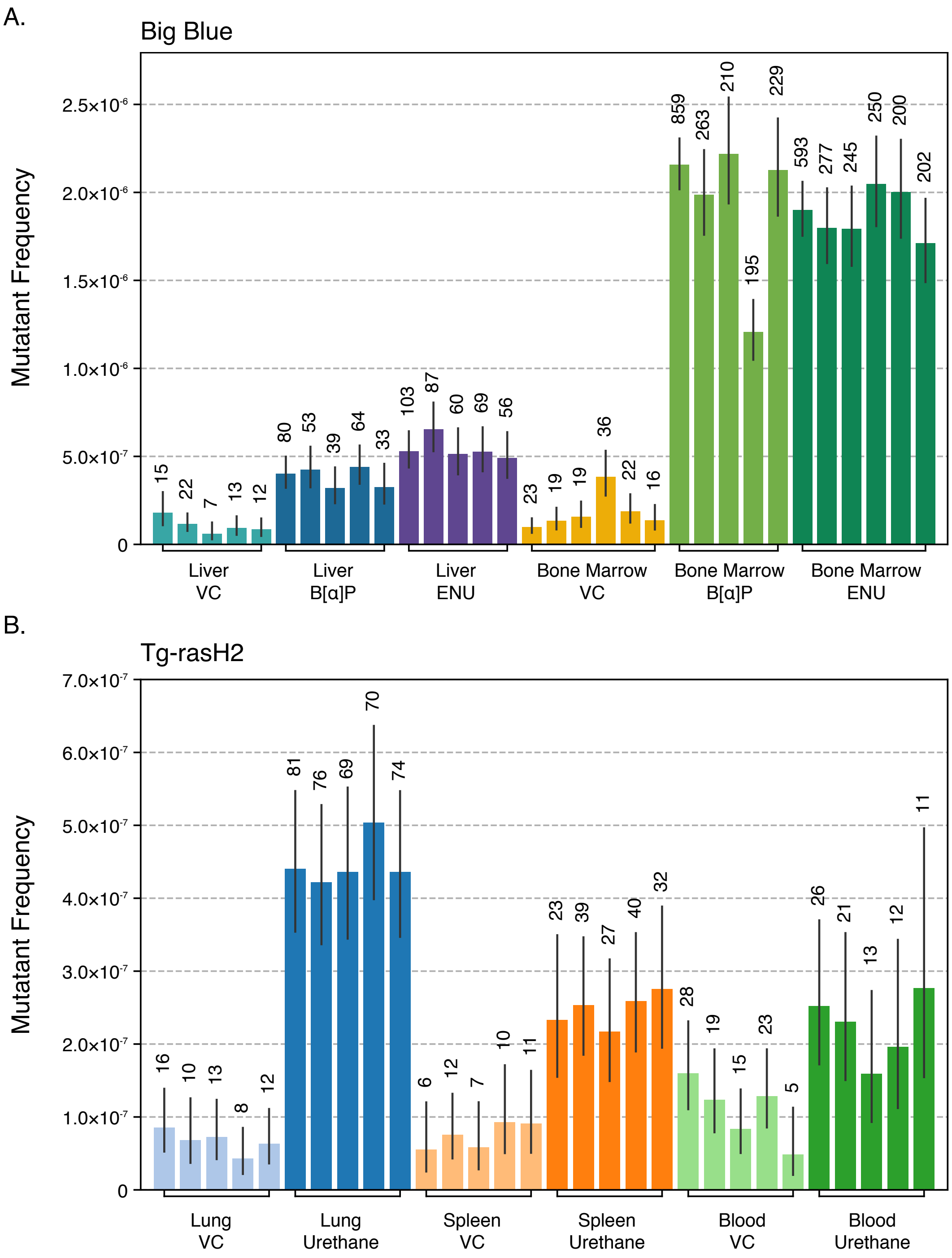

Figure S3. Per-sample mutant frequencies for all tissues and treatment groups. A) Big Blue cohort samples. **B)** Tg-rasH2 cohort samples. Mutant frequency was calculated as the total number of non-germline mutant duplex consensus base pairs divided by the total number of duplex consensus base pairs per sample. Error bars reflect 95% binomial confidence intervals. Integers above each bar represent the total number of mutant duplex consensus alleles observed per sample. VC, Vehicle Control.

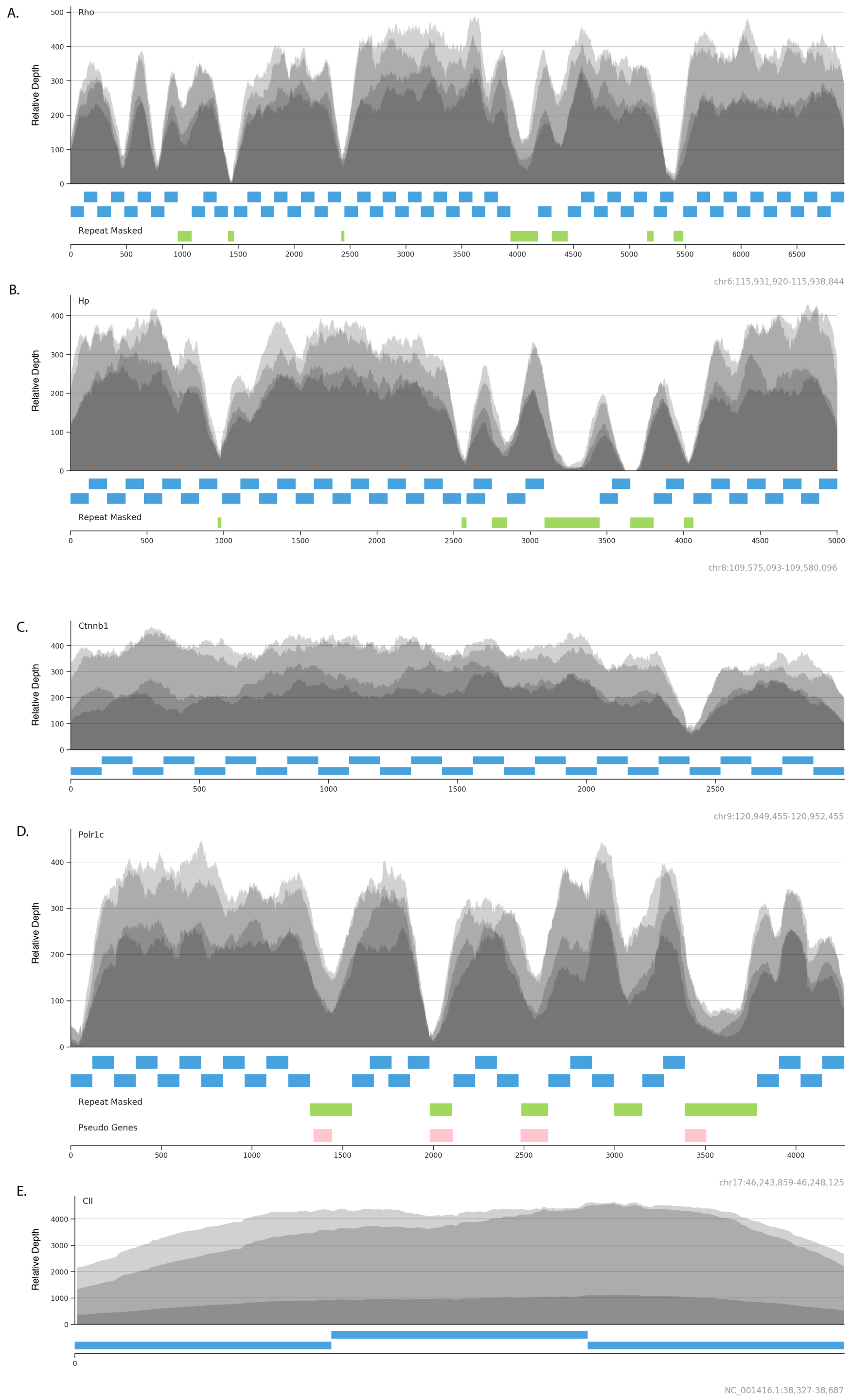

Figure S4. Consensus alignment data and probe design over endogenous and transgenic targets in the Big Blue mouse. Hybrid selection targets were carefully designed to abut no closer than 10 base pairs from a repeat masked (green) or pseudogene (pink) intervals. Individual baits are colored as blue intervals underlying the read coverage track. The four coverage tracks shown in all panels are from four randomly selected library preparations to illustrate the relatedness of coverage profile and bait layout. **A)** Example coverage and panel design over *Rho*, **B)** *Hp*, **C)** *Ctnnb1*, **D)** *Polr1c* and **E)** the Big Blue mouse *cII* transgene.

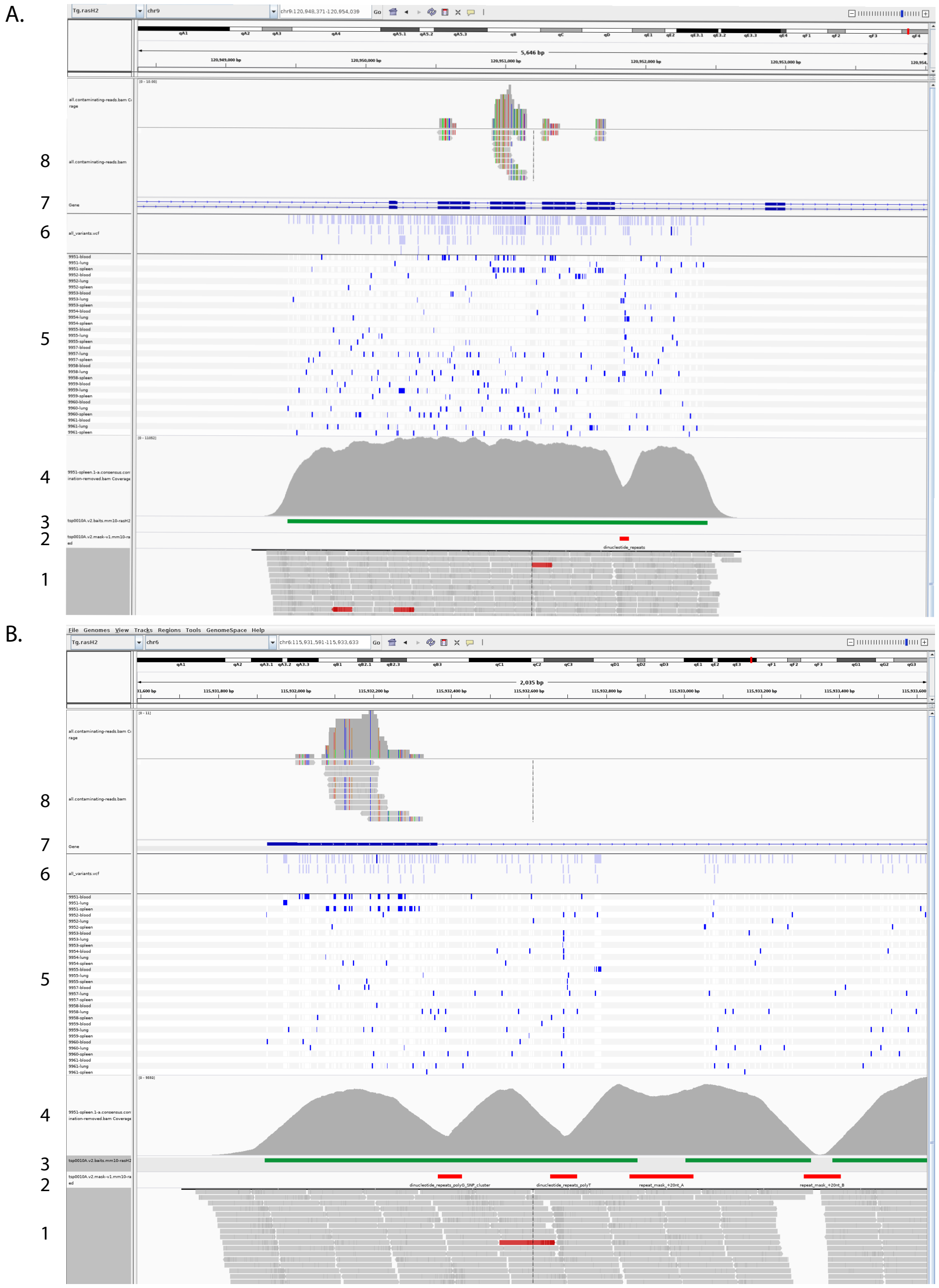

Figure S5. Ultra-rare contamination is easily detected by Duplex Sequencing but can be filtered from alignment data. Contamination of homologous species DNA drastically increases observed apparent mutations and confounds experimental frequency results in absence of identification and removal. **A**) A genomic view of the *Ctnnb1* gene in the transgenic mouse Tg-rasH2 (mm10). Tracks from bottom to top (1) consensus paired-end read alignments (2) repeat-masked regions (3) baited region (4) density of consensus paired-end read alignments (5) unfiltered variant calls for all samples (6) silhouette of unfiltered variant calls for all samples (7) transcript diagram of *Ctnnb1* (8) reads identified as contamination from all samples. **B**) A genomic view of the *Rho* gene in the transgenic mouse Tg-rasH2 (mm10). Tracks from bottom to top (1) consensus paired-end read alignments (2) repeat-masked regions (3) baited region (4) density of consensus paired-end read alignments (5) unfiltered variant calls for all samples (6) silhouette of unfiltered variant calls for all samples (7) transcript diagram of *Rho* (8) reads identified as contamination from all samples

SUPPLEMENTARY TABLES

Treatment	Tissue	Big Blue Mouse ID	No. of Phage Screened	No. of Mutant Phage	Mutant Frequency ($\times 10^{-6}$)
Vehicle Control	Liver	9301	795,000	32	40.3
		9302	479,000	24	50.1
		9303	851,667	36	42.3
		9304	1,005,000	37	36.8
		9305	1,090,000	57	52.3
		9306	1,878,333	75	39.9
	Bone Marrow	9301	1,056,667	43	40.7
		9302	883,333	15	17.0
		9303	945,000	27	28.6
		9304	814,667	30	36.8
		9305	452,333	22	48.6
		9306	1,122,333	64	57.0
Benzo[α]pyrene	Liver	9307	1061,667	183	172.4
		9308	533,333	140	262.5
		9309	1,048,333	212	202.2
		9310	73,8333	159	215.3
		9311	898,333	161	179.2
	Bone Marrow	9307	461,667	359	777.6
		9308	636,667	438	688.0
		9309	982,167	563	573.2
		9310	480,000	295	614.6
		9311	486,667	356	731.5
N-ethyl-N-nitrosourea	Liver	9313	603,333	129	213.8
		9314	268,000	62	231.3
		9315	1,091,667	211	193.3
		9316	926,667	128	138.1
		9318	764,333	121	158.3
	Bone Marrow	9313	1,310,000	413	315.3
		9314	1,443,333	509	352.7
		9315	1,081,667	336	310.6
		9316	1,120,000	501	447.3
		9317	555,333	279	502.4
		9318	966,667	486	502.8

Table S1: Summary of *cII* mutant, and total, phage counts from Big Blue mouse samples assayed via the transgenic rodent assay.

Treatment	Tissue	Mouse ID	Duplex Base Pairs	Duplex Read Pairs	Contaminant Read Pairs
VC	Liver	9301	83,347,487	447,345	3
		9302	189,664,560	1,073,461	
		9303	116,151,844	644,555	
		9305	139,635,456	758,419	
		9306	142,517,573	788,174	
	Marrow	9301	233,209,849	1,299,710	
		9302	142,676,998	816,171	
		9303	122,126,416	691,682	
		9304	93,720,485	516,633	
		9305	117,074,653	647,591	
		9306	116,291,270	654,545	
BaP	Liver	9307	199,781,219	1,116,103	1
		9308	124,921,520	690,952	
		9309	121,889,164	668,854	2
		9310	145,497,107	787,576	28
		9311	101,239,854	558,976	
	Marrow	9307	398,180,336	2,211,892	
		9308	132,469,507	737,436	
		9309	94,683,250	534,719	
		9310	161,559,440	908,313	
		9311	107,702,271	598,033	
ENU	Liver	9313	194,351,371	1,094,930	
		9314	133,182,703	734,197	
		9315	117,123,025	649,237	
		9316	131,236,780	728,655	
		9318	114,044,868	624,536	
	Marrow	9313	311,985,917	1,754,334	
		9314	154,012,541	857,890	
		9315	136,572,030	757,932	
		9316	122,129,838	711,785	
		9317	99,925,780	536,425	
		9318	118,085,724	680,974	
**Total**			**4,716,990,836**	**26,282,035**	**34**

Table S2. Sequencing summary of the Big Blue mouse samples including consensus duplex bases and read pairs assayed. Almost 5 billion duplex bases were generated from 26 million duplex consensus read pairs. Only 34 read pairs were positively assigned to either the *Rattus norvegicus albus* or *Homo sapiens* species and were removed prior to variant calling.

Treatment	Tissue	Mouse ID	Duplex Base Pairs	Duplex Read Pairs	Contaminant Read Pairs
VC	Blood	9951	207,394,262	1,086,331	10
		9952	182,197,208	968,700	
		9953	213,403,886	1,082,589	1
		9954	211,788,461	1,122,824	
		9955	122,203,569	672,834	
	Lung	9951	223,122,766	1,147,311	
		9952	174,477,089	915,140	
		9953	214,233,783	1,099,934	1
		9954	221,040,720	1,140,582	
		9955	225,338,412	1,159,455	
	Spleen	9951	128,760,653	679,168	19
		9952	189,196,859	982,779	2
		9953	142,603,705	755,718	
		9954	127,428,088	660,354	
		9955	142,475,597	755,444	
Urethane	Blood	9957	120,633,024	685,489	
		9958	106,729,358	597,808	
		9959	95,461,826	564,675	
		9960	71,696,388	442,693	
		9961	46,755,705	326,118	
	Lung	9957	219,224,515	1,144,532	
		9958	214,603,131	1,107,643	
		9959	188,293,412	961,323	
		9960	164,292,248	838,850	
		9961	201,809,864	1,039,777	
	Spleen	9957	116,655,635	621,762	
		9958	183,345,724	935,859	
		9959	146,584,262	776,730	
		9960	183,685,874	954,588	
		9961	138,129,660	708,501	
**Total**			**4,348,868,321**	**25,982,044**	**33**

Table S3. Sequencing summary of the Tg-rasH2 mouse samples including consensus duplex bases and read pairs assayed. Similar to the experimental design for sequencing of the Big Blue mouse samples, nearly 5 billion duplex base pairs were generated from 26 million duplex consensus read pairs from the Tg-rasH2 sample set. From these samples, 33 contaminating read pairs were detected from both *Rattus norvegicus albus* and *Homo sapiens* species. These reads were removed prior to downstream analysis.

Mouse ID	Tissue	Treatment	Variant Allele Depth	Total Allele Depth	Variant Allele Frequency
9957	Lung	Urethane	320	17,644	1.8136%
9958	Lung	Urethane	189	17,477	1.0814%
9959	Lung	Urethane	61	14,686	0.4154%
9961	Lung	Urethane	17	15,854	0.1072%
9961	Spleen	Urethane	2	10,809	0.0185%
9951	Blood	VC	0	16,218	
9952	Blood	VC	0	14,198	
9953	Blood	VC	0	17,094	
9954	Blood	VC	0	16,626	
9955	Blood	VC	0	10,072	
9957	Blood	Urethane	0	9,862	
9958	Blood	Urethane	0	8,788	
9959	Blood	Urethane	0	8,536	
9960	Blood	Urethane	0	7,232	
9961	Blood	Urethane	0	5,763	
9951	Lung	VC	0	17,658	
9952	Lung	VC	0	13,040	
9953	Lung	VC	0	17,126	
9954	Lung	VC	0	17,742	
9955	Lung	VC	0	18,150	
9960	Lung	Urethane	0	11,886	
9951	Spleen	VC	0	9,073	
9952	Spleen	VC	0	14,586	
9953	Spleen	VC	0	9,794	
9954	Spleen	VC	0	8,878	
9955	Spleen	VC	0	9,824	
9957	Spleen	Urethane	0	7,541	
9958	Spleen	Urethane	0	14,597	
9959	Spleen	Urethane	0	10,444	
9960	Spleen	Urethane	0	14,590	

Table S4. Early neoplastic evolution is detected with Duplex Sequencing in the cancer-predisposed mouse Tg-rasH2. The variant allele counts of A·T→T·A mutations at codon 61 in the human *HRAS* transgene in the Tg-rasH2 mouse model. The variant allele counts observed at this locus are those of A·T→T·A in the context CTG for urethane exposed tissues. All but one urethane exposed lung tissue harbors a variant at significant clonality. A single urethane exposed splenic sample has a small clone of two counts (0.018%) at this locus.

SUPPLEMENTARY DATA FILES

Database S1. Tabular text file of all variant calls for the Big Blue samples in MUT format.
Database S2. Tabular text file of all variant calls for the Tg-rasH2 samples in MUT format.

DUPLEX READ DATA FILES

Final filtered and decontaminated error-corrected alignments for all 62 mouse samples in the BAM file format are deposited in the Sequence Read Archive under BioProject accession PRJNA673916.

REFERENCES

1. Wood, D. E. & Salzberg, S. L. Kraken: ultrafast metagenomic sequence classification using exact alignments. *Genome Biol.* **15**, 1–12 (2014).
